# PF4 in rejuvenation therapy: Neuroprotection and cognitive enhancement

**DOI:** 10.17305/bb.2025.11960

**Published:** 2025-04-01

**Authors:** Li Li, Chunming Xie

**Affiliations:** 1 Center of Health Management, Affiliated ZhongDa Hospital, School of Medicine, Southeast University, Nanjing, Jiangsu, China; 2 Department of Neurology, Affiliated ZhongDa Hospital, School of Medicine, Southeast University, Nanjing, Jiangsu, China

**Keywords:** Platelet factor 4, PF4, anti-aging, neuroinflammation, cognitive impairment, rejuvenation

## Abstract

Platelet factor 4 (PF4), a platelet-derived chemokine found in the blood, has been identified as a critical factor in modulating the rejuvenation of the aged brain. Increasing evidence suggests that PF4 secretion is a prerequisite for the cognitive benefits associated with young blood transfusion, the longevity factor klotho, and exercise. Systemic administration of exogenous PF4 has been shown to reduce circulating pro-aging immune factors and restore peripheral immune function in the aged brain by mitigating age-related hippocampal neuroinflammation, promoting molecular changes in synaptic plasticity, and improving cognitive function in aged mice. Clinically, reduced serum PF4 levels have been significantly associated with cognitive decline and core pathological biomarkers in Alzheimer’s disease. Mechanistically, the chemokine receptor CXCR3 partially mediates the cellular, molecular, and cognitive benefits of systemic PF4 administration in the aged brain. However, several critical questions remain, including the potential role of PF4 in blood–brain communication, its interaction with neurotransmitters and neuropharmacological processes, and how these findings might be translated into clinical practice. Further detailed studies are needed to validate and expand upon these insights for therapeutic application.

## Introduction

With age, everyone will inevitably face the reality of brain aging, including a decline in memory and learning ability. It is commonly accepted that systemic aging profoundly influences our high-order emotional and cognitive abilities and substantially contributes to the development of aging-related diseases, thereby leading to dementia. As such, identifying therapeutics for systemic rejuvenating interventions to reverse age-related impairments is a real dream for humans. Although the underlying cause of aging is the irreversible cessation of cell division and entry into a permanent state of growth arrest without undergoing the process of cell death, the gradual functional deterioration of the immune system, known as immune aging, is critical for modulating the aging process [1]. Specifically, increased neuroinflammation levels in aged mice or humans can lead to the persistence of immune-related aging cells and the continuous secretion of many proinflammatory factors, thereby poisoning surrounding cells, driving the aging process, and contributing to cognitive impairment [2]. These senescent cells ultimately result in age-related diseases, such as diabetes, cancer, Alzheimer’s disease (AD), and atherosclerosis. Intriguingly, rejuvenation research has found that injecting blood from young individuals into the aging brain could significantly ameliorate and potentially reverse age-related cognitive decline in aged mice or humans [2–5]. However, the underlying mechanisms at the cellular and molecular levels in the aging process, especially the critical component responsible for rejuvenating the aged brain, remain largely unknown.

## PF4 as a critical anti-aging factor

Fortunately, a series of recent studies have identified that platelet factor 4 (PF4), as a critical anti-aging component, could delay brain aging and even return the aging brain to youth. For example, multiple studies have found that injecting blood from young individuals into elderly mice could enhance their motor ability and restore their aging brain function to younger levels [3, 6, 7]. At the molecular level, a longevity factor, also called klotho, has been reported to improve memory and cognitive abilities in elderly individuals [8]. Additionally, simple exercise was also identified to delay cognitive decline and reduce the risk of neurodegenerative diseases, although it cannot reverse aging [2, 7]. These beneficial effects of young blood, the “longevity factor” klotho, and exercise on improving cognitive ability all relied on a chemotactic factor generated by platelets—PF4—in a series of randomized and blinded animal experiments [8–10]. Originally, the secretion of PF4 reversing brain aging existed in the overlooked components of blood. In fact, over the past 20 years, accumulating evidence has found that connecting the circulatory system of young and elderly mice could improve the brain function of elderly mice and relieve aging symptoms of multiple organ tissues, such as muscles, liver, heart, and bones [2, 3]. Professor Villeda’s group previously found that simply injecting young (three-month-old) individuals’ plasma into elderly mice (18–20 months) could transfer this beneficial effect [3], while blood plasma with platelets from young mice systematically exposed to aged male mice could substantially increase adult neurogenesis and brain-derived neurotrophic factor expression, reduce hippocampus-related neuroinflammation, and improve cognitive performance [1, 2]. Recent studies have found that plasma PF4 levels in young individuals were significantly higher than those in elderly mice or humans [10–13]. Importantly, when exogenous PF4 was injected into elderly mice, age-related hippocampal neuritis significantly improved and exhibited cellular and molecular changes related to synaptic plasticity, as well as showing better performance in various memory and learning tasks. In fact, PF4 makes the immune system look younger by reducing active anti-aging immune factors and neuroinflammation, increasing hippocampus-dependent synaptic plasticity, and ultimately enhancing cognitive flexibility [5, 14]. For example, a recent study reported that proanthocyanidins, as a dietary supplement, could substantially improve systemic inflammation, raise levels of the anti-inflammatory cytokine PF4, and significantly lower pro-inflammatory factors in the blood to rescue cognitive impairment in aging mice [15]. Patients with essential thrombocythemia often present with elevated platelets, which may increase the risk of thrombotic events and produce higher levels of PF4 [16]. Importantly, PF4 could balance hematological conditions to protect against immune aging and thrombotic events, maintaining life expectancy [16]. In addition, recent studies reported that decreased serum PF4 levels significantly correlated with cognitive decline and CSF levels of β-amyloid (Aβ)42 and t-tau in AD patients [11], and PF4 combined with seven other proteins could benefit early identification of AD patients [17]. Accordingly, these findings support the proposal that PF4, as a critical anti-aging component, could modulate age-related cognitive impairment in aged mice or AD patients.

## Mechanistic insights and open questions

**Figure 1.**
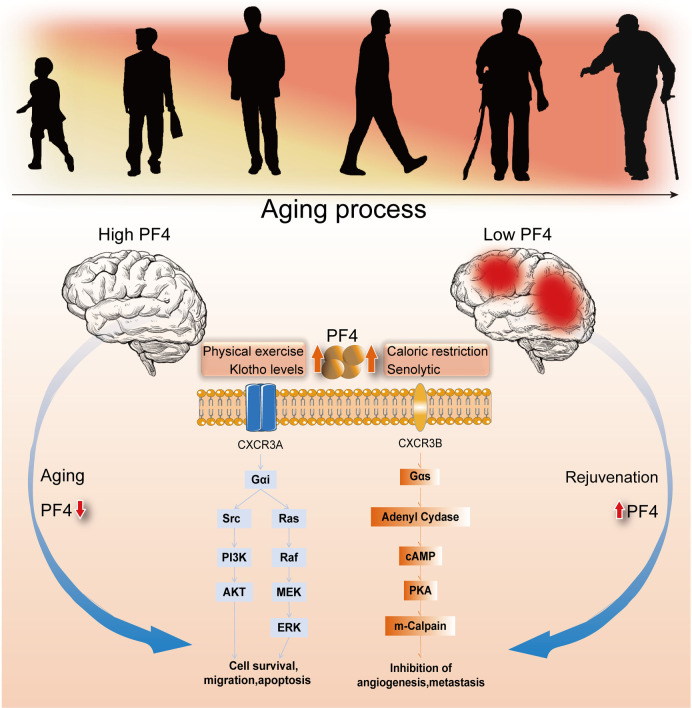
**Molecular mechanism of PF4 was involved in the rejuvenation research.** PF4: Platelet factor 4.

Mechanistically, although the exact molecular pathways or interactions by which PF4 works synergistically with other proteins or alone remain unclear, a growing body of evidence has identified that CXCR3, as a chemokine receptor, plays a critical role in mediating these beneficial effects of systemic PF4 administration in the aged brain [10, 18, 19]. More importantly, the PF4-CXCR3 complex can trigger multiple signaling pathways in distinct cell types. For example, the PF4-CXCR3 complex could activate the PI3K/AKT/Nrf2 or MEK/ERK pathways to mitigate age-associated immune dysfunction and hematopoietic diseases by affecting cell survival, proliferation, migration, and apoptosis [18, 19]. Alternatively, the PF4-CXCR3 complex could also increase cAMP production and mediate PKA and m-calpain activation to inhibit angiogenesis or metastasis [20]. However, the downstream signaling pathways of the PF4-CXCR3 complex in rejuvenation remain to be defined. The potential mechanism of the PF4-CXCR3 complex is described in Figure 1.

Nevertheless, some essential issues still should be further elucidated.

First, it is well known that systemic inflammation, coagulopathy, and neurovascular dysfunction often occur concurrently in neuropathological diseases [21]. However, the potential role of PF4 in blood–brain crosstalk, which represents the interactive effects of neutrophils, platelets, and neutrophil extracellular traps in the neuropathological process of aging, remains unclear. In fact, emerging evidence highlights that PF4 could orchestrate extensive cellular activation of neutrophils, platelets, monocytes, and endothelial cells through various mechanisms [22], and elevated PF4 substantially increases the risk of coagulation via mechanisms, including heparin-induced thrombocytopenia (HIT) and immune-mediated thrombosis. Specifically, complement activation probably participates in pathogenic HIT with higher anti-PF4 polyclonal levels, suggesting that complement activation represents a functional biomarker for platelet-activating antibodies in HIT. In addition, Wang et al. [23] reported in a clinical case published in the New England Journal of Medicine that vaccine-induced immune thrombocytopenia and thrombosis associated with coronavirus disease 2019 is related to PF4 antibodies in a heparin-independent manner; however, the exact mechanism remains unclear. As such, we should exercise caution regarding coagulation when additional PF4 is administered as a promising therapeutic agent. Moreover, the adverse effects of PF4 administration leading to fibrosis [24] and neuronal ferroptosis in cerebral hemorrhage [18] should be noted. Neutralizing the PF4-glycosaminoglycans interaction may relieve the progression of fibrosis, while activating the CXCR3/PI3K/AKT/Nrf2 pathway could mitigate hemorrhage-induced neuronal ferroptosis.

Second, the beneficial effects of PF4 administration in reversing the aged brain to a young state depend on hippocampus-related neurogenesis and synaptic plasticity, especially in the dentate gyrus, not in the cortex or cerebellum [2, 9], subsequently accelerating memory and learning improvement in the aged brain. However, accumulating evidence has demonstrated that improved memory and learning ability is tightly linked with the activity of neurotransmitters or specific neural circuits. Future studies still need to investigate how PF4 influences the neurotransmitter system and neuropharmacological modulation of NMDA, noradrenaline, and endocannabinoid receptors or interacts with specific neural circuits implicated in memory and learning [25–27]. Additionally, a recent report argued that PF4 levels depend on an individual’s age; that is, higher levels of PF4 in plasma were observed in young individuals compared to older individuals [13]. However, another study found that PF4 levels were determined by the donor’s age, with elderly donors exhibiting elevated PF4 levels compared to younger donors [28]. This discrepancy may be due to activated platelet concentration and the recruited subjects’ age [28].

Third, PF4 displays good power as a diagnostic biomarker for age-related cognitive decline and identifies its crucial role in the increased incidence of dementia-related disorders, especially in AD patients. In fact, Sun et al. [11] identified that significantly decreased serum PF4 levels were positively correlated with cognitive decline and CSF biomarkers, including reduced Aβ40 and Aβ42, and negatively correlated with increased total tau proteins in Aβ-positive AD patients in a Chinese cohort, indicating that PF4 may become a promising anti-aging and therapeutic target for AD. However, it is unclear whether serum PF4 levels could be detected early in the preclinical stage of the AD spectrum population, especially in patients with subjective cognitive impairment and mild cognitive impairment. As such, the dynamic trajectory of PF4 levels should be mapped in the AD spectrum population, and more participants in the clinical cohort must be recruited to validate these findings. In addition, as Sun [11] mentioned, the area under the curve of serum PF4 was weaker compared to those of CSF Aβ42, ptau181, and t-tau in AD patients, while a large percentage of serum PF4 levels from the original data overlapped between healthy controls and AD patients [11]. These findings indicate that PF4 integrated with other potential biomarkers or therapeutic targets may provide a more powerful tool to differentiate AD patients from healthy controls [17].

Fourth, building on recent progress, we propose that serum PF4 levels in the AD spectrum should be measured along with Aβ40, Aβ42, total and phosphorylated tau proteins, and even tau217 and tau181, because the latter two represent early, sensitive biomarkers for the identification of an AD-related high-risk population [29–35]. Then, we should determine whether PF4 levels correlate with cognitive performance and pathological biomarkers of AD at each stage, as described in the publication [11]. Additionally, as a therapeutic target, monitoring changes in serum PF4 levels induced by pharmacological or neuromodulation therapy should be tracked in clinical practice [36, 37]. Of course, more attention should be paid to dosage optimization, potential side effects, and ethical concerns surrounding long-term administration of PF4 in clinical practice. Although the off-target binding of an anti-Aβ monoclonal antibody to PF4 causes acute and chronic toxicity in cynomolgus monkeys [38], the manner in which PF4 dominantly modulates Aβ deposition and subsequently attenuates cognitive impairment deserves further clinical investigation. More importantly, it is necessary to track dynamic changes in plasma Aβ and tau protein levels, as core biomarkers of AD, and explore the potential mechanism underlying PF4-driven molecular changes and cognitive improvement when PF4 is administered to the AD spectrum population. However, trans-species differences may limit the generalizability of preclinical translation of exogenous PF4 administration.

## Conclusion

Overall, recent studies have demonstrated that increasing systemic levels of PF4 in the cerebrovasculature could ameliorate age-related neurodegeneration and cognitive impairment in a hippocampal neurogenesis-dependent manner. PF4 interacting with CXCR3 may represent a promising molecular signaling pathway, potentially crucial for balancing the inhibition of angiogenesis or metastasis and promoting cell survival, proliferation, and migration to protect against age-induced cognitive impairment and rejuvenate aged immune systems. In clinical practice, selecting PF4 as a therapeutic target may delay or rescue cognitive decline in old age by inhibiting the neuroinflammatory response. As a peripheral blood biomarker, PF4 is easy to detect and cost-effective, which may compensate for the invasive limitations of CSF testing. More importantly, identifying the potential physiological processes and signaling pathways by which PF4 targets the molecular and cellular mechanisms underlying cognitive function is conducive to developing novel PF4 therapeutic agents in the future.
